# The role of identity in the DSM-5 classification of personality disorders

**DOI:** 10.1186/1753-2000-7-27

**Published:** 2013-07-31

**Authors:** Klaus Schmeck, Susanne Schlüter-Müller, Pamela A Foelsch, Stephan Doering

**Affiliations:** 1 Child and Adolescent Psychiatric Hospital, Psychiatric University Hospitals, Basel, Switzerland; 2 Practice for Child and Adolescent Psychiatry, Frankfurt, Germany, and University of Applied Sciences FHNW, Basel, Switzerland; 3 Weill Medical College of Cornell University, New York, USA; 4 Department of Psychoanalysis and Psychotherapy, Medical University of Vienna, Vienna, Austria

**Keywords:** DSM-5, Personality disorder, Identity, Adolescence, Classification

## Abstract

In the revised Diagnostic and Statistical Manual DSM-5 the definition of personality disorder diagnoses has not been changed from that in the DSM-IV-TR. However, an alternative model for diagnosing personality disorders where the construct “identity” has been integrated as a central diagnostic criterion for personality disorders has been placed in section III of the manual. The alternative model’s hybrid nature leads to the simultaneous use of diagnoses and the newly developed “Level of Personality Functioning-Scale” (a dimensional tool to define the severity of the disorder). Pathological personality traits are assessed in five broad domains which are divided into 25 trait facets. With this dimensional approach, the new classification system gives, both clinicians and researchers, the opportunity to describe the patient in much more detail than previously possible. The relevance of identity problems in assessing and understanding personality pathology is illustrated using the new classification system applied in two case examples of adolescents with a severe personality disorder.

## Introduction

The emergence of the self in childhood and adolescence is based on experience and perception, which then becomes organized into identity, which organizes further experience and perception. Identity is related to the individual’s “selfsameness and continuity in time” [1], and the others’ recognition of these qualities also. Experience is constituted by the subjective, emotional “I” while perception is the basis of coherence and the definitory “Me” [2]. Understanding the development of identity from a psychological perspective and how it is integrated in the new DSM-5 classification system are the focus of this paper. In two case examples we will illustrate impairment of identity integration in adolescent patients with personality disorders (PD).

In their developmental considerations for the new DSM system Tackett and colleagues [3] describe a life span perspective of personality pathology from early childhood to later life. In spite of the reluctance of many clinicians to use the diagnosis before the age of 18, there is a constantly growing body of evidence that PDs can be diagnosed already in adolescence [4-6]. Personality pathology seems to be highest before the age of 20, with a decline of most of the pathological features (especially in the Cluster B domain) over time [7]. The diagnostic criteria of both, ICD-10 and DSM-IV-TR, define personality disorders to begin in childhood or adolescence. DSM-5 states cautiously that “Personality disorder categories may be applied with children or adolescents in those relatively unusual instances in which the individual’s particular maladaptive personality traits appear to be pervasive, persistent, and unlikely to be limited to a particular developmental stage or another mental disorder.” ([8], p. 647). If symptoms of Borderline Personality Disorder (BPD) are assessed already in early adolescence [6], the prevalence rate of BPD in an epidemiological sample of 11 year old children was 3.2%. Reliability, validity, and temporal stability of BPD-diagnoses in adolescents are similar to those in adulthood [9,10].

The use of PD diagnoses before adulthood is of high importance for the development of therapeutic approaches that can address this special kind of pathology with developmentally appropriate therapeutic techniques. Along with the higher acceptance of PD diagnoses in adolescents in the last decade there is substantial progress of specific psychotherapies for adolescents by adapting approaches that had been developed for adult populations. Currently five manualized approaches for the therapy of adolescents with personality disorders are available: Dialectical Behavior Therapy DBT-A [11], Cognitive-Analytic Therapy (CAT) [12], Emotion Regulation Training for Adolescents (ERT) [13], Mentalization Based Treatment (MBT-A) [14] and Adolescent Identity Treatment (AIT) [15].

### From DSM-IV to DSM-5

During the development of the current revision of the Diagnostic and Statistical Manual of Mental Disorders (DSM-5) [8], that has been published in May 2013, there was discussion to change the classification of personality disorders (PD) from that in the DSM-IV-TR. The rationale for a substantial change referred to six arguments [16]:

1. Extensive co-occurrence among PDs (having one PD diagnosis is associated with a high risk to fulfil the criteria of other PD diagnoses).

2. Extreme heterogeneity among patients receiving the same diagnosis (e.g. 256 different ways to diagnose a Borderline PD).

3. Lack of synchrony with modern medical approaches to diagnostic thresholds.

4. Temporal instability (inconsistent with the relative stability of personality traits).

5. Poor coverage of personality psychopathology (Personality Disorder not otherwise specified is the most frequently diagnosed PD in clinical practice).

6. Poor convergent validity (an indicator of major difficulties to clearly operationalize the criteria).

The weight of these arguments is compelling and would indicate that a substantial change to the DSM-IV system is warranted. However, due to political controversies, the American Psychiatric Association (APA) Board of Trustees decided in December 2012 that the DSM-5 would maintain the categorical model and the criteria for the 10 personality disorders as it had been in DSM-IV-TR (for an overview see the issue of the Journal of Personality Disorders, Dec 2012). All proposed changes, including the new trait-specific methodology, were moved to a separate area of the DSM-5 Section III titled “Alternative DSM-5 Model for Personality Disorders” (8), where proposals were placed that require further research.

The Board of Trustees decided to keep the old criteria (with well-known lack of reliability and validity) despite the major revision proposed by the DSM-5 Personality Disorders Work Group. The proposed new classification system was based on 14 years of work, was more evidence-based, and with potential for greater clinical (and research) utility than DSM-IV. The major focus of the proposed revision was on the introduction of a dimensional model to the assessment and understanding of personality disorders, parallel to the dimensional models of normal personality that are widely accepted. Since there is still a need for categorical diagnoses in our current health care system, the Work Group proposed a hybrid model of personality disorders. In addition to the requisite categorical approach of DSM-IV, a dimensional approach was included to assess pathological personality trait domains and trait facets as well as a “Level of Personality Functioning-Scale” as an overall measure of the severity of personality dysfunction [17]. However, the decision of the APA Board of Trustees retains a 30 year old system that remains in substantial need of repair.

The next years will reveal if clinicians and researchers will continue to use the DSM-IV-TR system or if they will start to use the hybrid model of DSM-5 Section III. The new proposal has already stimulated research activities (see for example the April 2013 issue of the journal ‘Assessment’). From our point of view the revision will be of high clinical and scientific value, especially in adolescent patients where the dimension of functioning captures the nuances of development more accurately.

### Diagnosing personality disorders in DSM-5 Section III

The core criteria of a personality disorder are seen as significant impairments in self and interpersonal functioning that are assumed to be continuously distributed. In the DSM-5 Section III conceptualization of personality disorders, self-functioning is defined by the two constructs of “identity” (does a person experience him- or herself as unique, with clear boundaries between self and others?) and “self-direction” (how is a person able to pursue goals in life and to self-reflect productively), whereas interpersonal functioning is based on “empathy” and “intimacy”(is a person able to understand other people’s perspectives and form close relationships?).

In addition, the diagnosis of PD can only be made if pathological personality traits are present in at least one of five broad domains: negative affectivity, detachment, antagonism, disinhibition vs. compulsivity, and psychoticism.

With the new “Levels of Personality Functioning Scale” (Table 1) five levels of impairment can be differentiated on a continuum of severity ranging from “no impairment” (level 0) to “extreme impairment” (level 4).

**Table 1:** DSM-5 Level of Personality Functioning Scale (APA, 2013; p. 775–777)

	**Self**		**Interpersonal**	
**Level**	**Identity**	**Self-direction**	**Empathy**	**Intimacy**
**0** Little or no im-pairment	- Has ongoing awareness of a unique self; maintains role-appropriate boundaries- …	- Sets and aspires to reasonable goals based on a realistic assessment of personal capacities**-** …	- Is capable of accurately understanding others’ experiences and motivations in most situations- …	- Maintains multiple satisfying and enduring relationships in personal and community life- …
**1** Some impairment	- Has relatively intact sense of self, with some decrease in clarity of boundaries when strong emotions and mental distress are experienced**-** …	- Is excessively goal-directed, somewhat goal-inhibited, or conflicted about goals.**-** …	- Is somewhat compromised in ability to appreciate and understand others’ experiences; may tend to see others as having unreasonable expectations or a wish for control.**-** …	- Is able to establish enduring relationships in personal and community life, with some limitations on degree of depth and satisfaction.**-** …
**2** Moderate impairment	- Depends excessively on others for identity definition, with compromised boundary delineation.**-** …	- Goals are more often a means of gaining external approval than self-generated, and thus may lack coherence and/or stability.**-** …	- Is hyperattuned to the experience of others, but only with respect to perceived relevance to self.**-** …	- Is capable of forming and desires to form relationships in personal and community life, but connections may be largely superficial.**-** …
**3** Severe impairment	- Has a weak sense of autonomy/agency; experience of a lack of identity, or emptiness. Boundary definition is poor or rigid: may show overidentification with others, overemphasis on independence from others, or vacillation between these.**-** …	- Has difficulty establishing and/or achieving personal goals.**-** …	- Ability to consider and understand the thoughts, feelings and behavior of other people is significantly limited; may discern very specific aspects of others’ experience, particularly vulnerabilities and suffering.**-** …	- Has some desire to form relationships in community and personal life is present, but capacity for positive and enduring connections is significantly impaired.**-** …
**4** Extreme impairment	- Experience of a unique self and sense of agency / autonomy are virtually absent, or are organized around perceived external persecution. Boundaries with others are confused or lacking.**-** …	- Has poor differentiation of thoughts from actions, so goal-setting ability is severely compromised, with unrealistic or incoherent goals.**-** …	- Has pronounced inability to consider and understand others’ experience and motivation.**-** …	- Desire for affiliation is limited because of profound disinterest or expectation of harm. Engagement with others is detached, disorganized or consistently negative.**-** …

### DSM-5 general criteria for personality disorders (APA, 2013, p.761)

The essential features of a personality disorder are:

A. Moderate or greater impairment in personality (self /interpersonal) functioning.

B. One or more pathological personality traits.

C. The impairments in personality functioning and the individual’s personality trait expression are relatively inflexible and pervasive across a broad range of personal and social situations.

D. The impairments in personality functioning and the individual’s personality trait expression are relatively stable across time, with onsets that can be traced back to at least adolescence or early adulthood.

E. The impairments in personality functioning and the individual’s personality trait expression are not better explained by another mental disorder.

F. The impairments in personality functioning and the individual’s personality trait expression are not solely attributable to the physiological effects of a substance or another medical condition (e.g., severe head trauma).

G. The impairments in personality functioning and the individual’s personality trait expression are not better understood as normal for an individual’s developmental stage or sociocultural environment.

Using the proposed DSM-5 model the following standard approach to the assessment of personality pathology has been recommended [17].

### Standard approach to the assessment of personality pathology according to DSM-5

1. Is impairment in personality functioning present or not?

2. If so, rate the level of impairment in self and interpersonal functioning on the “Levels of Personality Functioning Scale”.

3. Is one of the 6 defined personality disorder types present?

4. If so, record the type and the severity of impairment.

5. If not, is a “Personality Disorder - Trait Specified” (PD-TS) present?

6. If so, record PD-TS, identify and list the trait domain(s) that are applicable, and record the severity of impairment.

7. If a PD is present and a detailed personality profile is desired and would be helpful in the case conceptualization, evaluate the trait facets.

8. If neither a specific PD type nor PD-TS is present, evaluate the trait domains and/or the trait facets, if these are relevant and helpful in the case conceptualization.

The twenty-five specific trait facets that are grouped in the five higher order personality trait domains (negative affectivity, detachment, antagonism, disinhibition vs. compulsivity, psychoticism) are used to “compose” the categorically defined PDs as well as those that have been eliminated. As an example the trait-based description of the borderline personality disorder is given here:

Pathological personality traits in the following domains:

1. **Negative Affectivity**, characterized by:

a. **Emotional lability:** Unstable emotional experiences and frequent mood changes; emotions that are easily aroused, intense, and/or out of proportion to events and circumstances.

a. **Anxiousness:** Intense feelings of nervousness, tenseness, or panic, often in reaction to interpersonal stresses; worry about the negative effects of past unpleasant experiences and future negative possibilities; feeling fearful, apprehensive, or threatened by uncertainty; fears of falling apart or losing control.

a. **Separation insecurity:** Fears of rejection by – and/or separation from – significant others, associated with fears of excessive dependency and complete loss of autonomy.

a. **Depressivity:** Frequent feelings of being down, miserable, and/or hopeless; difficulty recovering from such moods; pessimism about the future; pervasive shame; feeling of inferior self-worth; thoughts of suicide and suicidal behavior.

2. **Disinhibition,** characterized by:

a. **Impulsivity:** Acting on the spur of the moment in response to immediate stimuli; acting on a momentary basis without a plan or consideration of outcomes; difficulty establishing or following plans; a sense of urgency and self-harming behavior under emotional distress.

a. **Risk taking:** Engagement in dangerous, risky, and potentially self-damaging activities, unnecessarily and without regard to consequences; lack of concern for one’s limitations and denial of the reality of personal danger.

3. **Antagonism,** characterized by:

a. **Hostility:** Persistent or frequent angry feelings; anger or irritability in response to minor slights and insults ([8], p.766-767).

### Personality functioning and the dimension of identity

The assessment of personality functioning goes back to the psychoanalytic concept of personality structure. Kernberg [18] was the first to combine the domains of identity, psychic defences, and reality testing to distinguish different levels of personality functioning or – in his terms – level of personality organization (i.e. neurotic, borderline, & psychotic). In Kernberg’s view, the core pathology of patients with borderline and other severe personality disorders can be found in an impairment of their identity integration, what he called identity diffusion. His basic assumption is that due to innate predispositions to aggression and/or adverse childhood experiences, internalized aspects of the self and significant others are not integrated into whole (integrated positive and negative) internal images (“representations”) of the self and significant others [19]. Clinically, this state of identity diffusion leads to severe difficulties in describing oneself and others as well as problems in developing a sense of self with attitudes, interests, and life goals that are stable and reliable over time. Another consequence of identity diffusion occurs in the realm of interpersonal relationships. Due to their fragmented representations of others, borderline patients are characterized by an impaired ability to mentalize, to empathize, and to build up and rely on stable relationships. Particularly intimate relationships are burdened by frequently changing self-states and either idealized or devaluated views of the partner [19].

For the assessment of personality organization (i.e., personality functioning) in borderline patients, Kernberg developed the Structural Interview [20], a clinical interview that considerably influenced later diagnostic instruments. For research purposes Clarkin et al. constructed the Structured Interview of Personality Organization (STIPO [21]), that assesses the domains described by Kernberg in a structured manner. The format of the 87 items resembles the Structured Clinical Interview for DSM-IV (SCID; [22,23]). A number of interviews and questionnaires have been developed during the last three decades that cover different aspects of personality functioning. Reviews of these instruments have recently been published by Bender et al. [24] and Doering & Hörz [25]. A new self-report instrument with promising psychometric properties has been developed for the assessment of identity pathology in adolescents ([2,26,27] this issue).

The clinical observation that the level of personality functioning is strongly associated with prognosis and outcome of psychiatric patients has repeatedly been confirmed empirically. Three studies employing the structure axis of the Operationalized Psychodynamic Diagnosis (OPD-[2,28]), a psychodynamically informed multi-axial diagnostic interview, predicted a worse treatment outcome in patients with impaired personality structure [29-31]. Recently Hopwood et al. [32] demonstrated that severity of personality disorders, defined in terms of the number of fulfilled diagnostic criteria of all DSM-IV PDs, significantly correlates with social, work, and leisure dysfunction. Preoccupation with social rejection, fear of social unskillfulness, feelings of inadequacy, anger, identity disturbance, and paranoid ideation loaded most highly on the dimension of severity of impairment. In addition to a lack of capacity for intimacy and pro-social behaviour, Livesley [33] describes the lack of stable and integrated representations of self and others as the third core factor of general personality dysfunctioning. Skodol et al. [34] reported that a five-item screening for personality disorders solely covering aspects of identity integration predicts the presence of a PD with a sensitivity of 79% and a specificity of 54% (if more than 3 out of 5 items were coded with “yes”). The five items used in this study on 424 psychiatric patients were:

1. I can hardly remember what kind of person I was only a few months ago.

2. My feelings about people change a great deal from day to day.

3. Most of the time I don’t have the feeling of being in touch with my real self.

4. I drift through life without a clear sense of direction.

5. I have very contradictory feelings about myself [34].

In the light of these results, the selection of the domains of identity and interpersonal functioning as a measure for severity in the DSM-5 Levels of Personality Functioning Scale appears reasonable and empirically supported.

### Identity development in adolescents

The consolidation of identity is one of the most central developmental tasks of adolescence. Erikson [1] formulated the concepts of normal ego identity, identity crisis, and identity diffusion as the crucial characteristics of normal and pathological personality development.

Identity crisis is a period of lack of correspondence between the view of the adolescent by his immediate environment derived from the past, in contrast to the adolescent’s relatively rapid changing self-experience that, at least transitorily, no longer corresponds to others’ view of him or her [35]. Thus, identity crisis derives from a lack of confirmation by others of the adolescent’s changing identity. This normal identity crisis, however, must be differentiated from identity diffusion, the pathology of identity characteristic for borderline patients and other severe personality disorders.

Erikson [1] described identity diffusion as an absence or loss of the normal capacity for self-definition, reflected in emotional breakdown at times of physical intimacy, occupational choice, and competition, and increased need for a psychosocial self-definition. He suggested that the avoidance of choices reflecting such identity diffusion led to isolation, a sense of inner vacuum, and regression to earlier identifications.

Identity diffusion would be characterized by the incapacity for intimacy in relationships, because intimacy depends on self-definition, and its absence triggers the sense of danger of fusion or loss of identity that is feared as a major calamity. According to Erikson, identity diffusion is also characterized by diffusion of the time perspective, reflected either in a sense of urgency regarding decision making or in a loss of regard for time in an endless postponement of such decision making. Identity diffusion also shows in the incapacity to work creatively and in breakdown at work.

One central consequence of identity diffusion is the incapacity, under the influence of a peak affective state, to assess that affective state from the perspective of an integrated sense of self. The particular mental state may be fully experienced in consciousness, but cannot be put into the context of one’s total self-experience. This implies a serious loss of the normal capacity for self-reflection, particularly for mentalization [36], producing difficulties in differentiating the source of the affect, its meaning, or determining subsequent appropriate interaction in the reality. The structural condition of identity diffusion, in short, implies a significant limitation of the process of mentalization, and, under conditions of a peak affect state, a balanced and integrated representation of self and other are not possible. Identity diffusion [18,19] becomes the core of personality pathology resulting in decreased flexibility and adaptability of functioning in the area of self-regulation, interpersonal relations, and meaningful productive actions. These are assessed in the DSM-5 by means of the “Levels of Personality Functioning” [17] in the realm of “self ” and “interpersonal”.

We will now illustrate the relevance of the diagnostic criterion of identity pathology by means of two case vignettes of adolescent patients that are classified according to DSM-5.

### Case examples

#### Case 1

A mother brought her 17 year old daughter into treatment because the daughter seemed to be totally dependent on a boy who treated her very badly. The adolescent met this boy via the internet the year before and after only a short time, she wanted to move in with him (he lived 220 km away from her). Surprisingly the mother agreed to this plan, but the problem of changing to another school stopped the decision.

In the first meeting I saw a shy, quiet, mousy adolescent. She reported that she always was shy, didn’t like to speak in front of many people (e.g. in front of the class) and blushed. She was afraid of many people and preferred to be alone. On the other hand she reported she was absolutely dependent on other people and did not have the heart to do things alone. She described her relationship with her boy-friend as submissive (“humbleness for love”). On one hand she wanted him to be very near, and on the other hand she felt very scared about this nearness. She said she wanted him to be “part of me” and called him up to 20 times a day, not understanding how much she annoyed him, even when he threatened to leave her if she would not stop. When she could reach neither him nor her mother, she developed panic attacks and experienced dissociation and derealization.

She was not able to describe herself in an adequate way, using short and unelaborated descriptors (e.g. “I am shy, I need my boyfriend, I go to school”), had no coherent picture of herself (e.g. “I have no idea who I am”, “I only go to school and wait for the next day”), showed a lack of coherence (i.e. no capacity to be alone, suggestibility, no differentiation from others without feeling alone e.g. “I only want to be near my boy-friend or my mother”) and lack of continuity (i.e. no idea of the future and little connection to her past).

Her father was unknown; her mother was from the former German Democratic Republic (GDR, former communist part of Germany). The mother reported a childhood history in which she was separated very early from her own mother (after 3 weeks in a day nursery), leaving her feel insecure about “how to be a mother” herself. She was often beaten by her father and not protected by her mother “who had no empathy”. Despite the father’s abuse, she reported “The relation to my father was even better than to my mother, to her I didn’t even have one”.

Between 12 and 14 years of age, a teacher sexually abused the mother. After she confided in someone it became a scandal, because this teacher was a very high positioned officer in the “Stasi” (secret service of the GDR) and it became a big disadvantage to the family. In 1998, shortly before the fall of the wall in Berlin, they left the GDR.

The mother met the father of her daughter in Western Germany. He had a conduct disorder, so she left him early after the childbirth and brought up her daughter alone. She reported that in the first years she constantly thought about giving her daughter up for adoption because “I wanted to spare her my life of suffering”. She could not remember where her child was when she was hospitalised. Her daughter was placed in foster care at the age of 7 due to the multiple psychiatric inpatient treatments.

In the reality context of multiple separations from her mother, the daughter said she was extremely scared that the mother would give her away forever and when she was returned to the mother, she did everything to avoid this (i.e. was very quiet, honest, well-behaved and easy-going). This contributed to the history of separation anxiety since childhood, as she always was afraid that the mother would give her away. She gave the example; “I was always picked up last from kindergarten and was always afraid that she will not come”. When her mother brought her to foster care she thought it was a punishment and wondered about what she had done wrong.

She reported a suicide attempt 2 years prior to this consult. This was after a history of suicidal ideation since primary school, when she left little notes all over the flat in which she wrote, “I do not want to live anymore”. (The mother confirmed she had found those notes from her young daughter, but that she didn’t know how to react and therefore she did not react at all). The daughter described herself as, “I think that I already was a sad baby”. She reported 3 previous psychotherapeutic treatments, which she dropped out of, and a trial of medication (SSRI) without any improvement.

##### Discussion of case 1

The adolescent described above has severe problems in self and interpersonal functioning. Her description of herself is superficial, vague and unelaborated (“I am shy, I need my boyfriend, I go to school”) despite her intelligence (IQ 120). She shows severe depressive symptoms and separation anxiety from childhood until the present. She reports dissociative symptoms (“I cannot feel my body anymore”, “I see myself from the outside, like in a movie”). The adolescent has a very unstable and incoherent picture of herself (“I have no idea who I am”, “I only go to school and wait for the next day”), her identity is severely disturbed (no capacity to be alone, suggestible, no differentiation from others without feeling alone, self- description is empty and only related to what her boyfriend or mother does, no perspective). Her interpersonal relationship is only to stabilize her feelings of deep loneliness; it is exchangeable (it doesn’t matter if it is the mother or boyfriend who is present, the main thing is that a person is available). She does not enjoy intimacy with her boy-friend, and the relationship has a sado-masochistic tone (“humbleness for love”). She has no idea of the impact of her behavior on her boyfriend, who is extremely annoyed by her constant calls (no empathy).

##### Psychosocial background of case 1

There was a severe and chronic disruption of the relationship with the mother that interfered with bonding (during the first years the mother wanted to give her up for adoption). The mother herself suffered from severe psychiatric problems, as well as physical and sexual abuse in her childhood. The daughter experienced repeated and long lasting separations from her mother in early childhood (while the mother did not even remember where her child was when she was hospitalized) (Table 2).

**Table 2:** **Summary: DSM**-**5 classification of case 1**

**General criterion**	**Patient suffers from severe personality impairment**	
**Level of impairment in self and interpersonal functioning** (0=not disturbed; 4=extremely disturbed)	**Levels of personality functioning scale**	
	- Identity: 4	- Empathy: 4
	- Self-direction: 3	- Intimacy: 3
**Personality disorder type**	**Personality disorder-trait specified**	
**Trait domains** (0=not disturbed; 3=extremely disturbed)	Negative affectivity: 3	Detachment: 0
	Antagonism: 0	Psychoticism: 0
	Disinhibition: 0	
**Trait facets** (0=not disturbed; 3=extremely disturbed)	**Facets of the trait domain “Negative affectivity”**	
	Emotional lability: 1	Anxiousness: 3
	Separation insecurity: 3	Perseveration: 3
	Submissiveness: 3	Hostility: 0
	Depressivity: 3	Suspiciousness: 2

#### Case 2

Patient is a 15 year old boy who was brought to treatment by his parents because of “laziness” regarding school work, “disobedience” within the home (e.g. not following the rules regarding his diet, exercise or TV/Video game time), as well as lying (e.g. about homework completion, food eaten, TV/Video time, but also money missing), conflicts with siblings (e.g. envy at perceived favoritism resulting in dismissive, critical or aggressive verbiage), mood (e.g. oscillations from irritability or sadness, to elation or excitement), and long standing attentional problems (e.g. distractibility or perseveration). He was easily hurt by the perceived criticisms of others, had difficulties in social skills evidenced in a limited number of friends, few invitations to other children’s parties, and being a target for bullying. However, with little awareness of the hurt, he responded to perceived attacks with arrogance and devaluation of others. His teachers also reported that his arrogant and prideful behavior provoked his peers.

He was originally brought for consultation at age 9 for inattention, distractibility and difficulties completing tasks in school. At this time, it was also reported that he had an “obsession” with food and eating. For example, prior to going to an event he would ask (with an anxious tone and need for reassurance) what food would be available there. He presented with a low activity level (parents described this as his having an “engine on idle”) and resistant to almost any change. Parents and he would engage in “negotiations” to do or change things. He had pronounced self-esteem issues, constantly putting himself down and berating himself for poor performance in school (e.g. even when he received a 97% on a spelling test). Psychological testing indicated an intelligent boy, with reading and decoding skills in the superior range, but with a weakness in writing, attention, and executive functioning. The parents sought treatment with a psychiatrist for the attentional problems and school difficulties. He was treated with 54 mg Methylphenidate and had regular therapy sessions, until he “stopped talking”. He was then brought to a Social Skills group, but no improvement was observed.

Parents return for this evaluation, reporting he “is showing a complete and total lack of motivation” by not doing his homework, not studying, lying about it, etc. The parents are frustrated as “he claims to have goals but won’t do anything to achieve them”. The mother reports she has “had enough”, is hopeless that things can change, and has “no more energy” to invest in helping him. The father “has not given up” and is trying to fill in the “extra attention” that mom is not providing, but then feels resentful of the mom and the son. School performance is significantly impaired and the family tensions are very high, with constant arguments between him and his parents, and tensions with the two younger siblings who compete for parent’s attention. Although objectively, he received a lot of attention, he had no feeling of gratefulness because he was convinced that everything was due him (entitlement). When asked about the impact on his siblings, he was dismissive of their concerns and spoke in a callous way.

There is a family history of mood disorder, attentional problems, and Obsessive Compulsive disorder on both sides.

The son presents appearing younger than his age, overweight, with the look of “baby fat”. He does not understand that his parents are concerned about him and want to help him. Instead, he described their hopelessness that things can change with bitter contempt and sarcasm. His report minimizes the consequences of his poor school performance and he is convinced that he can succeed. He says he understands what he has to do to perform the tasks and achieve the goals, but is not willing to sustain or take productive action. He says he “just hates school”, and explains his lack of motivation because “school is of no use to him”. When questioned “how” he thinks he can succeed, he explains that his father will call the school and talk with the teachers to get extensions or reductions in the work. He sees no contradiction between his insistence he can do the work while having no sense of having to invest in his own actions, and his simultaneous reliance on his father to negotiate less work for him. The poor self-esteem is defended against by grandiosity regarding his abilities, while at the same time he relies on others for help. His descriptions of important others was affected by obvious envy, which he however in reverse described as their envy of him.

Taken together this indicates a lack of “integrity of self-concept” defined in the DSM-5 Levels of Personality Functioning as “Regulation of self-esteem and self-respect; sense of autonomous agency; accuracy of self-appraisal; quality of self-representation” [34].

At home, he reports daily conflicts with both parents, but particularly the mother, who chastises his food choices. He hoards food, sneaks it into his room, leaves the empty containers in his room and then denies having eaten the food (despite the evidence in plain view). Food is often used to bribe him to participate or complete activities that the parents require (e.g. school work, going to the tutor, etc.). The pattern of negotiating and demanding as well as taking action only toward his immediate goal (contrary to expected) and then lying or denying this, are now chronic and pervasive, characteristic of manipulativeness and deceitfulness (aspects of “antagonism”) His behavior is singularly motivated and he is unable to integrate this into the expectations of others or his own long-term goals indicative of problems in “Self-directedness” as well as difficulties in the “Interpersonal” realm, especially with respect to “Empathy” or “Intimacy and cooperativeness” [34].

His self-description demonstrates a lack of “identity integration” [34,37], when he speaks in vague and impressionistic terms, oscillating between grandiose statements of his intellectual capacities and plans to go to an Ivy League college, and self-deprecating statements of inferiority, inability to perform or complete tasks well. This also illustrates his inability to make links between his past, the present and his future, speaking in a disconnected way. When he describes his difficulties with his weight, he focuses not on the problem of his overeating and poor food choice (a real health concern as he has been medically diagnosed as pre-diabetic), but on how his parents “hide” the snack food, and “won’t let him” eat Chinese food. He emphases how “mean” his parents are because they force him to run on the tread mill while he watches TV, instead of just being able to “relax”. He distorts the reality in the service of feeling like the victim, without recognizing the reality of his own behavior (lack of self-control and motivation) that had provoked the parent’s “incentives” program. This view indicates a problem in the “Complexity and integration of representations” of others [34].

When asked to describe a friend, he hesitates, unable to think of a person to describe. When pushed, he identifies one boy, younger than himself, who he plays video games with online. There is no depth to the description, “He plays games with me”, and no indication of the relationship as being anything other than of convenience (he belongs to their community group and the parents are friends). He also described preferring to spend time with adults, as “they like me better”. He reported difficulty in making or keeping friends as a result of how they just see “how special” he is and were envious of him and aspire to be like him.

##### Discussion of case 2

This adolescent presents criteria for a narcissistic PD, reacting to criticism with anger and shame, imaging unrealistic fantasies of success, power and intelligence and in setting unrealistic goals. He appears unemotional and requires constant attention from his parents and teachers without any empathy regarding their feelings. He is obsessed with himself, easily hurt and becomes jealous easily. Due to this, it is impossible for him to keep healthy relationships to his parents, peers or even siblings. In addition to these presenting difficulties, initial testing showed weakness in executive functioning and difficulty in integration of affect. These processing weaknesses are associated with problems in regulatory aspects of personality functioning [38]. Despite treatment for the attentional and social difficulties with standard psychopharmacotherapy and behavioral social skills training, these regulatory and organizational processes which are related to personality, showed a decline over the 6 years as observed in the current significant functional difficulties in school, family and with peers. Additional issues within the family, the conflict between mom and dad over the image of the child (e.g. his physical image, weight especially), the shifting attribution of “blame” and “responsibility”, and the maintenance of the “negotiating” strategy of regulating action compound the difficulties of this boy. As can be seen in the clinical description, this boy has significant impairments in the areas of self (problems in identity integration, integrity of self-concept and self-directedness) and interpersonal (problems in empathy, intimacy and cooperativeness, and a lack of complexity and integration of representations of others). His difficulties indicate a need for a specialized treatment that focuses on development of identity integration and differentiation (clarifying the interaction between himself and his family) (Table 3).

**Table 3:** **Summary: DSM**-**5 classification of case 2**

**General criterion**	**Patient suffers from severe personality impairment**	
**Level of impairment in self and interpersonal functioning** (0=not disturbed; 4=extremely disturbed)	**Levels of personality functioning scale**	
	- Identity: 4	- Empathy: 3
	- Self-direction: 3	- Intimacy: 4
**Personality disorder type**	**Narcissistic personality disorder**	
**Trait domains** (0=not disturbed; 3=extremely disturbed)	Negative affectivity: 3	Detachment: 2
	Antagonism: 3	Psychoticism: 0
	Disinhibition: 2	
**Trait facets** (0=not disturbed; 3=extremely disturbed)	**Facets of the trait domain “Negative affectivity”**	
	Emotional lability: 2	Anxiousness: 1
	Separation insecurity: 0	Perseveration: 3
	Submissiveness: 1	Hostility: 3
	Depressivity: 3	Suspiciousness: 2
	**Facets of the trait domain “Detachment”**	
	Restricted affectivity: 2	Withdrawal: 3
	Intimacy avoidance: 3	Anhedonia: 2
	**Facets of the trait domain “Antagonism”**	
	Manipulativeness: 3	Deceitfulness: 3
	Grandiosity: 3	Attention Seeking: 2
	Callousness: 0	
	**Facets of the train domain “Disinhibition/compulsivity”**	
	Irresponsibility: 3	Impulsivity: 3
	Distractability: 3	Risk Taking: 1
	Rigid perfectionism: 0	

## Conclusions

The new DSM-5 classification system has been published in May 2013. The changes that were intended to be made in the personality disorders diagnoses in comparison to DSM-IV were remarkable and covered many areas. However, these changes have been moved to an appendix, the so called Section III of the DSM-5. The main diagnostic criteria remained unchanged. In comparison to a single diagnosis the amount of information that is given within the complete diagnostic procedure of this newly proposed classification system is enormous, what is demonstrated with two cases examples of a 17 year old girl and a 15 year old boy. The diagnosis of the girl in the first case vignette would be “dependent PD” in DSM-IV-TR and DSM-5. In the alternative model of the DSM-5 system there are four stages of assessment instead. First, it can be stated that the girl suffers from substantial personality impairment. Second, the level of impairment in self and interpersonal functioning is described as severe impairment in the four areas of identity, self-directedness, empathy and intimacy. The diagnostic label of “PD-Trait Specified” that is assigned in the third step is elucidated by the assessment on five broad trait domains and 25 more specific trait facets, the fourth step (the diagnosis “dependent PD” has been skipped in this system due to the lack of empirical evidence). This broad assessment gives a lot of information that characterizes the patient in much more detail and thus can give many hints for treatment planning.

The boy of the second case example suffers from a narcissistic PD, but this diagnosis alone would not really characterize his broad personality pathology that is already consolidated at the age of 15. An abnormal development can be seen in four of the five trait domains (negative affectivity, antagonism, detachment and compulsivity), and the description on the trait facets clarifies the clinical picture in much more detail. For example, within the domain of “disinhibition vs. compulsivity” the particular pattern of facets, including irresponsibility (e.g. lack of regard for completing homework or following the rules of the house to not eat in his room), distractibility (i.e. his difficulty in maintaining goal-focused behaviour), and rigid perfectionism (e.g. preoccupation with specific details and order of things) support the diagnosis of narcissism. More importantly, it shows the particular characteristics that comprise how the narcissism manifests in this boy and the level of severity. The ratings also permit changes in the pattern and levels to be monitored over the course of treatment.

Both patients show clear signs of identity diffusion, one of the main characteristics of severe personality disorders. In both cases the identity pathology cannot be seen as a part of normal “adolescent turmoil” as it can be found in identity crises. The identity diffusion shows up in a lack of continuity in the experience of self and others and a lack of a coherent self that can be derived from contradictory behaviour and insufficient capacity for cognitive self-reflection. In both the boy and the girl these signs can be traced back to late childhood or early adolescence and are stable over time. This is characteristic for adolescents that present with severe personality disorders at a very early time of their development. Treatment approaches have to bear in mind this identity pathology that has to be addressed in order to arrive at a long-lasting change of the disorder. The new psychotherapeutic approach Adolescent Identity Treatment (AIT); [15,39] has been developed to place identity pathology in the focus of treatment.

We have explicitly decided not to present cases of Borderline Personality Disorders as frequently personality pathology in adolescents is seen synonymous with Borderline pathology, especially if identity impairment is present. However, as is described in the alternative DSM-5 classification, identity diffusion is not only a core symptom of Borderline Personality Disorder, but is one of the central features of all personality disorders.

Already before publication DSM-5 in general has been under severe debate as it is judged as the “bible” of psychiatry that defines the boundary between normality and mental illness [40]. With such a definitional power a classification system like DSM-5 transcends the limits of a medical handbook and achieves a societal influence that is far beyond its original scientific basis. The major point of discussion is the lowering of thresholds to define a mental disorder which can lead to an enormous increase of the prevalence of a certain disorder from one day to the other. The potential consequences of this approach can be found in the new definition of personality disorders in the alternative model in section III of DSM-5. While the definition in section II refers to “**clinically significant** distress or impairment in social, occupational, or other important areas of functioning” ([8]; p. 646), the authors of the alternative model in section III define as main general criterion (A) “**moderate** or greater impairment in personality (self/interpersonal) functioning” ([8]; p. 761). Interestingly, the authors of the DSM-5 personality disorder working group had proposed in 2011 that the threshold of the main criterion A should be “significant impairment” [17]. This substantial lowering of the threshold will have enormous (both positive and negative) effects if it will be implemented in clinical routine. The authors of DSM-5 have described their rationale for this shift of threshold: “Furthermore, the moderate level of impairment in personality functioning required for a personality disorder diagnosis in DSM-5 Section III was set empirically to maximize the ability of clinicians to identify personality disorder pathology accurately and efficiently”. [41] A low threshold is advantageous for a screening instrument in order to minimize the beta-error (false negative), or in clinical terms to be sure that no patient with a personality disorder is not detected. This is useful for offering the maximum amount of support to patients and leads to higher health care utilisation. For the use of such definition in an adolescent population one has to be quite critical about this low threshold, because the counterpart of a low beta-error is a high alpha-error, or in clinical terms: with such a low threshold there is a substantial chance to give a diagnosis of personality disorder to an adolescent that doesn’t have the disorder (false positive). We suggest that in adolescents the threshold should be higher (severe impairment) so that the diagnosis of a personality disorder is given more restrictively.

From our point of view the abrupt decision of the APA Board of Trustees to move the dimensional model of personality to section III and to keep the old model DSM-IV-TR in section II is unfortunate and disrupts progress in the field of both PD research and clinical practice. We are aware that the proposed DSM changes illustrated in this article are quite complex and it would take time and training for clinicians to fully understand and apply the new system. With the decision of the APA committee to dislocate the trait-specific methodology to a separate section, it will take some years and further research to decide if an image of personality pathology that is nearer to (dimensional) reality will be accepted.

On the other hand it is a major health policy issue that the proposed changes in DSM-5 could lead to a loosening of diagnostic thresholds with the unintended consequence of an inflation of diagnoses. Therefore it will be essential to use the new system with prudence in order to not to enlarge the definition of pathology to such an extent that is neither acceptable for society in general, nor helpful for clinical diagnosis -especially for those who are at the edge between personality pathology and an extreme personal style that should be accepted as part of human nature.

## Competing interests

The authors declare that they have no competing interests.

## Authors’ contributions

All authors have written parts of the manual and contributed substantially to the text. All authors read and approved the final manuscript.
